# Temporal Progression of Aortic Valve Pathogenesis in a Mouse Model of Osteogenesis Imperfecta

**DOI:** 10.3390/jcdd10080355

**Published:** 2023-08-20

**Authors:** Kaitlyn Thatcher, Carol R. Mattern, Daniel Chaparro, Veronica Goveas, Michael R. McDermott, Jessica Fulton, Joshua D. Hutcheson, Brian R. Hoffmann, Joy Lincoln

**Affiliations:** 1 Department of Pediatrics, Division of Pediatric Cardiology, Medical College of Wisconsin, Milwaukee, WI 53226, USA; kthatcher@mcw.edu (K.T.); cmattern@mcw.edu (C.R.M.); govever70@gmail.com (V.G.); 2 Herma Heart Institute, Children’s Wisconsin, Milwaukee, WI 53226, USA; 3 Department of Biomedical Engineering, Florida International University, Miami, FL 33174, USA; dchap015@fiu.edu (D.C.); jhutches@fiu.edu (J.D.H.); 4 Center for Cardiovascular Research, The Abigail Wexner Research Institute at Nationwide Children’s Hospital, Columbus, OH 43205, USA; mcdermott.mr1@gmail.com (M.R.M.); jessica.fulton@osumc.edu (J.F.); 5 Mass Spectrometry and Protein Chemistry, Protein Sciences, The Jackson Laboratory, Bar Harbor, ME 04609, USA; brian.hoffmann@jax.org

**Keywords:** aortic valve disease, extracellular matrix, connective tissue disorder

## Abstract

Organization of extracellular matrix (ECM) components, including collagens, proteoglycans, and elastin, is essential for maintaining the structure and function of heart valves throughout life. Mutations in ECM genes cause connective tissue disorders, including Osteogenesis Imperfecta (OI), and progressive debilitating heart valve dysfunction is common in these patients. Despite this, effective treatment options are limited to end-stage interventions. Mice with a homozygous frameshift mutation in *col1a2* serve as a murine model of OI (*oim/oim*), and therefore, they were used in this study to examine the pathobiology of aortic valve (AoV) disease in this patient population at structural, functional, and molecular levels. Temporal echocardiography of *oim/oim* mice revealed AoV dysfunction by the late stages of disease in 12-month-old mice. However, structural and proteomic changes were apparent much earlier, at 3 months of age, and were associated with disturbances in ECM homeostasis primarily related to collagen and proteoglycan abnormalities and disorganization. Together, findings from this study provide insights into the underpinnings of late onset AoV dysfunction in connective tissue disease patients that can be used for the development of mechanistic-based therapies administered early to halt progression, thereby avoiding late-stage surgical intervention.

## 1. Introduction

The heart maintains unidirectional blood flow throughout the cardiac cycle via four sets of valves (aortic, mitral, tricuspid, and pulmonic) that open and close over 100,000 times a day. The mitral and tricuspid valves are referred to as atrioventricular and consist of 2 or 3 valve leaflets, respectively, with external supporting chordae tendineae. In contrast, the aortic and pulmonic valves (semilunar) each have three leaflets referred to as cusps with internal supporting apparatuses. No matter the position, each leaflet or cusp is comprised of highly organized layers of extracellular matrix (ECM) arranged according to blood flow: the ventricularis, made predominantly of elastin, faces the inflow of blood and allows the leaflets to stretch into the closed position; the fibrosa, made predominantly of circumferentially aligned collagen fibers, is exposed to the outflow of blood and provides strength to the leaflets; and the spongiosa, made mostly of proteoglycans and glycosaminoglycans, lies in between the fibrosa and ventricularis to provide softness and compressibility to protect the valve from hemodynamic flow [1]. The valve ECM is secreted and maintained by a population of valve interstitial cells (VICs) that reside within the core of the leaflet/cusp, while valve endothelial cells (VECs) provide a protective layer covering the surface, and molecularly communicate with the VICs to coordinate ECM homeostasis [1,2,3]. Together, the composition and organization of the valve ECM is critical for maintaining optimal biomechanical properties and thus function throughout life.

According to 2021 American Heart Association statistics, the incidence of valvular heart disease is 64 per 100,000 person-years with aortic valve (AoV) disease being the most prevalent (47.2% stenosis, 18.0% regurgitation) [4]. In contrast to healthy valves, disease is characterized by an overall disorganization of the connective tissue within the cusp as a result of imbalanced ECM secretion, and/or excess degradation of healthy matrix, that can significantly impair biomechanical performance [1,5,6]. For example, defining features of myxomatous valve disease include an abnormal accumulation of proteoglycans and collagen fiber breakdown, which weakens function, resulting in prolapse and/or regurgitation of the valve leaflet/cusp. In contrast, an excess abundance of collagen fibers in the form of valvular fibrosis leads to tissue stiffening, limited leaflet/cusp movement, and functional stenosis (reviewed in [7]). Collectively, these pathological changes result in loss of structural–functional integrity which is often not detected until the end stage of the disease, when effective treatment options are often limited to surgical valve repair or replacement. Therefore, there remains a critical need to fully understand the temporal pathogenesis of valve disease in order to improve diagnosis, treatment options, and outcomes.

Manifestations of valve dysfunction are observed in patients affected by genetic mutations in ECM components that underlie connective tissue disorders, including Marfan syndrome, Ehlers–Danlos Syndrome, and Osteogenesis Imperfecta (OI). OI is a rare, inherited systemic connective tissue disease caused by mutations in *COL1A1* or *COL1A2* that lead to varying degrees of bone fragility based on the abundance of these collagen types in healthy osteogenic tissue [8]. Col1 is also highly expressed in heart valve leaflets/cusps, chordae tendineae, annuli fibrosi, and myocardial tissue, and therefore, cardiovascular diseases including valvular stenosis and/or regurgitation are prominent phenotypes in OI patients [9]. While the pathophysiology of skeletal defects in OI patients has been well reported, few studies have described the effects of decreased Col1 content on cardiovascular manifestations [10,11]. Insights of human OI phenotypes have been gained through the utilization of the *oim/oim* mouse model, which presents with skeletal fractures, limb deformities, generalized osteopenia, and overall small body size [12]. Since this original publication, independent studies have further reported cardiovascular defects in these mice, including altered myocardial mechanics [10] and structural thickening of the valves [11]. However, extensive temporal characterization studies are lacking.

The goal of this study was to use the *oim/oim* mouse model to develop a deeper understanding of valvulopathies in OI patients. The experimental design focuses on examining temporal pathological changes in the composition and organization of the AoV ECM using histology, immunofluorescence, and mass spectrometry analysis in combination with functional studies by echocardiography. For the first time, we characterize the structure–function relations of the AoV during the progression of OI pathogenesis caused by *Col1a2* deficiency. By identifying early versus late landmarks of disease, we can aid in the development of therapeutic targets to halt or prevent progression to end stage valve dysfunction, thereby avoiding surgical repair or replacement.

## 2. Materials and Methods

### 2.1. General Statistics

The a priori power calculation was applied prior to the study to determine the sample size needed in order to detect some level of effect with inferential statistics. Following this, the data were subjected to non-parametric one-way ANOVA or *t*-test, as indicated in figure legends, to determine statistical significance. All statistical analysis and the generation of graphs were performed using Prism GraphPad 9.0 or Microsoft Excel version 16. For graphs, the mean data are presented in addition to standard error of the mean. “n” is defined as biological replicates and indicated in the figure legends.

### 2.2. Mice

Heterozygous *B6C3Fe a/a-Col1a2oim/J* (*oim*/+) mice were purchased from The Jackson Laboratory (#001815) and bred together to obtain *oim/oim* (null), *oim*/+ (heterozygotes) and *wildtype* genotypes. Mice were fed standard laboratory diet and housed in a controlled environment with 12 h light/dark cycles at 21 °C and 23% humidity and water ad libitum. Animals were euthanized via CO_2_ exposure followed by secondary euthanasia via cervical dislocation (adult mice) at the indicated time points (3, 6, 9, and 12 months of age). All animal procedures were approved by The Medical College of Wisconsin (MCW) Institutional Animal Care and Use Committees (Protocol #AUA00006769). Sex was not observed to be a biological variable. Therefore, both male and female mice were used, and the data were pooled.

### 2.3. Immunocytochemistry and Immunofluorescence

For histology experiments, unless otherwise stated, hearts were collected from mice at relevant timepoints, fixed in 4% paraformaldehyde, processed for paraffin embedding, and sectioned at 7 μm. For all antibodies (see Table 1), paraffin was removed through a series of xylene treatments, and tissue sections were rehydrated through a graded ethanol series and rinsed in 1x PBS. Sections containing the AoV were then subjected to antigen retrieval by boiling for 10 min in unmasking solution (Vector Laboratories, Newark, CA, USA #H-3300). Following this, sections were treated for 1 h at room temperature in blocking solution (1% BSA, 1% cold water fish skin gelatin, 0.1% Tween-20/PBS) and incubated overnight at 4 °C or 1 h at room temperature with indicated primary antibodies diluted in blocking solution or a 1:1 solution of 1x PBS and blocking solution.

For primary antibody detection, sections were incubated for 1 h at room temperature with appropriate secondary antibodies including Alexa Fluor 488 or Alexa Fluor 568 donkey α-rabbit, donkey α-goat, and donkey α-mouse diluted in 1x PBS (1:400, LifeTechnologies, Carlsbad, CA, USA), then mounted in Vectashield anti-fade medium with DAPI (Vector Laboratories, Newark, CA, USA #H-1500) to detect cell nuclei.

#### 2.3.1. Collagen Hybridizing Peptide

For collagen hybridizing peptide (CHP) immunofluorescence, paraffin sections were prepared as described above. To detect complete or total collagen, tissue sections were subjected to antigen retrieval by boiling for 10 min in unmasking solution (Vector Laboratories, Newark, CA, USA #H-3300). Sections were blocked as described above for 1 h at room temperature. Next, a 15 µm dilution of 5-FAM conjugated CHP (Advanced BioMatrix, Carlsbad, CA, USA #5264) in 1x PBS was heated for 5 min at 80 °C and immediately cooled on ice before adding 50 µL of solution to each section and incubating overnight at 4 °C. Slides were mounted in Vectashield anti-fade medium with DAPI (Vector Laboratories, Newark, CA, USA #H-1500) to detect cell nuclei.

#### 2.3.2. Pentachrome Staining

Whole hearts were collected from 3-, 6-, and, 12-month old *wildtype* and *oim/oim* mice and prepared according to the above methods for paraffin embedding. Movat’s Pentachrome staining was performed on paraffin tissue sections at each time point according to the manufacturer’s instructions (Russel Movat, American MasterTech, Lodi, CA, USA #KTRMP), then mounted using VectaMount Permanent Mounting Medium (Vector Laboratories, Newark, CA, USA #H-5000).

#### 2.3.3. Quantification of Immunoreactivity

Immunofluorescence and CHP images were visualized using an EVOS M7000 (ThermoFisher Scientific, Waltham, MA, USA) imaging system and software at 10× or 20× magnification (indicated by scale bars). Image brightness and contrast were edited using Adobe Photoshop 2022 v23.5.5, keeping edits consistent across experimental replicates. For corrected total cell fluorescence (CTCF) measurements, the average background fluorescence from each image was subtracted from the integrated density (a measurement of pixel intensity normalized to area) of the selected fluorescent channel in the AoV region as measured by ImageJ to calculate average CTCF for each image.

### 2.4. Echocardiography

Baseline echocardiography was performed in *wildtype* and *oim/oim* mice at 3, 6, and 12 months of age. Anesthesia was induced in mice with 3% isoflurane with 1 L per minute oxygen and maintained with 2% isoflurane with 1 L per minute oxygen. Anesthetized mice were secured to a heating platform in the supine position, and chest hair was removed via chemical hair remover. A warm echocardiography gel was placed on the chest wall to enhance sound transduction, and a small echocardiographic sound wave transducer was placed on the anterior chest wall. ECG electrodes were placed in a standard limb configuration to monitor heart rate. Body temperature was monitored using a rectal probe and controlled with a heat lamp and heating platform as described above. Ultrasound images were obtained using a Visual Sonics Vevo 2100 imaging system at 30 MHz or a Visual Sonics Vevo 3100 imaging system at 30 MHz.

Heart rate, cardiac output, left ventricular end systolic volume, left ventricular end diastolic volume, left ventricular end systolic diameter, and left ventricular end diastolic diameter were determined from M-mode short axis views at the level of the papillary muscles. Aortic diameter was measured using M-mode in a PLAX view of the ascending aorta. E/A ratios were captured using pulse wave Doppler on flow over the mitral valve. AoV velocity was captured from a suprasternal notch view in a right high parasternal location for optimal interrogation of the AoV. Aortic regurgitation was assessed via color, and flow Doppler and was only considered positive if present in both modes. The entire echocardiography procedure lasted approximately 10–15 min, after which mice recovered from anesthesia with heat assistance and were returned to the animal care facility. If the echocardiogram occurred on the day of a sacrifice, mice were allowed to recover prior to tissue harvest. Echocardiography statistics were measured via one-way ANOVA with multiple comparisons.

### 2.5. ECM-Enriched Proteomics by Mass Spectrometry

For each sample, one entire AoV structure (aortic ring containing three cusps) was collected and flash frozen for three biological replicates collected from 3-month-old *wildtype* and *oim/oim* mice. Each sample was subject to a decellularization process for ECM enrichment, resulting in two fractions: (1) the decellularized ECM peptide mixture and (2) the supernatant fraction resulting from the decellularization process. To achieve this, AoVs were thawed on ice in 50 μL of hypotonic lysis buffer (10 mM Tris-HCl, pH7.4) for 30 min. Following the thawing procedure, a final concentration of 5× (0.5%) RapiGest SF Surfactant (Waters, Milford, MA, USA #186001861) in H_2_O was added to each of the samples for the decellularization step. Following the initial thaw, each sample was snap-frozen in liquid nitrogen for 15 min and thawed at 37 °C for 15 min on a thermomixer set to 700 rpm. This freeze–thaw process was repeated for a total of 3 cycles, after which each of the sample homogenates were subject to ice water bath sonication (80 Hz, 100% power) for 5 min of 30 s on and 30 s off. Following the complete decellularization procedure, the supernatant fraction was carefully removed from the AoV tissue and transferred to a new microcentrifuge tube for protein digestion or storage at −80 °C. Note that the supernatant fraction was not utilized in this study. Sample resuspension buffer (50 µL of 50 mM ammonium bicarbonate, pH 8.3, plus 0.5% Rapigest) was added back to the AoV for protein digestion or storage at −20 °C until further use; as the proteolytic digestion is part of the extracellular enrichment procedure, there was no protein quantification performed at this step.

As indicated above, prior to proteolytic digest of the samples, 50 µL of 50 mM ammonium bicarbonate, (pH 8.3, plus 0.5% Rapigest) was added to the AoV decellularized sample. Dithiothreitol was then added to each sample to a final concentration of 10 mM and incubated at 42 °C for 30 min on a thermomixer set to 700 rpm. Following the reduction step, samples were cooled to room temperature for alkylation by adding iodoacetamide to a final concentration of 15 mM. Samples were incubated in the dark at 25 °C on a thermomixer set to 700 rpm for 30 min. Following alkylation, 1 µg of sequence-grade modified trypsin (Promega, Madison, WI, USA #V511A) was added to each sample and incubated for 16 h (overnight) at 37 °C on a thermomixer set to 500 rpm. After the initial 16 h, 0.5 µg of sequence-grade modified trypsin was added and incubated for another 4 h. Once proteolytic digestion was complete, the reaction was quenched by adding 1% final concentration of trifluoroacetic acid to adjust the pH to 3–4. Quenched samples then proceeded to the peptide clean-up step using Millipore P10 C18 zip-tips (#ZTC18S096) according to manufacturer protocol. All peptide eluents were dried in a vacuum centrifuge for 20 min.

The dried peptide samples were reconstituted in 20 µL of 98% H_2_O/2% acetonitrile/0.1% formic acid by pipetting liquid onto the sides of the tubes a minimum of 10 times, vortexing for 30 s, and then incubating the sample on a thermomixer at 22 °C for 10 min set at 700 rpm. Following reconstitution, the samples were spun on a tabletop low--speed centrifuge for 60 s, and the supernatant was transferred to a mass spectrometry vial for analysis. Each sample was injected in duplicate (8 µL each) into the instrument. The samples were then analyzed using a Thermo Fisher Scientific (Waltham, MA, USA) Eclipse Tribrid Orbitrap mass spectrometer coupled to an UltiMate 3000 liquid chromatography system with an EASY-Spray column (PepMap RSLC C18, 2 µm, 100 Å, 75 µm x 25 cm) in the Mass Spectrometry and Protein Chemistry Facility in the Protein Sciences Service at The Jackson Laboratory. The method duration was 180 min with a flow rate of 300 nL/min. Buffer A consisted of 100% H_2_O with 0.1% formic acid, and buffer B consisted of 100% acetonitrile with 0.1% formic acid. The gradient for the 180 min run consisted of 98% A/2% B to 95% A/5% B (0–8 min), 95% A/5% B to 70% A/30% B (8–130 min), 70% A/30% B to 10% A/90% B (130–135 min), 10% A/90% B to 98% A/2% B (135–140 min), and then three cycles to 5% A/95% B to 98% A/2% B (repeat 3 times total every four minutes) followed by equilibration for 30 min at 98% A/2% B. The Eclipse Tribrid Orbitrap settings included peptide mode, a default charge state of 2, Orbitrap HCD—high load (greater than 500 ng), and a cycle time of 3 s. For the precursor spectra detection (MS1) the settings included: detector = orbitrap, orbitrap resolution = 120,000, scan range = 375–1500 m/z, maximum inject time (ms) = 50, AGC target = 400,000, microscans = 1, data type = profile, polarity = positive, monoisotopic precursor selection was applied, charge states included = 2–7, dynamic exclusion of a n = 1 for 30 s with a 10 ppm mass tolerance, and a minimum intensity threshold of 5 × 10^4^. Peptide fragment analysis (MS2) settings included: quadrupole isolation mode, isolation window = 1.6, collision energy (%) = 33 (fixed), activation type = HCD, detector type = orbitrap, orbitrap resolution = 30,000, maximum inject time = 54 ms, AGC target = 50,000, data type = centroid, and polarity = positive.

All analysis of the mass spectrometry data was performed in the Mass Spectrometry and Protein Chemistry Facility at The Jackson Laboratory. The database searching and comparisons tools were performed using Proteome Discoverer 2.5.0.400. During the processing step, individual Thermo .RAW files were searched against the Mus musculus database (sp_tr_incl_isoforms TaxID = 10090, v2021-02-04) using Sequest HT. Search parameters included trypsin digest, precursor mass tolerance of 20 ppm, fragment mass tolerance of 0.5 Da, and multiple dynamic modifications (carbamidomethyl = +57.021 Da on cysteine, oxidation of methionine = +15.995 Da, acetylation of lysine/arginine = +42.011). The maximum number of missed cleavages allowed was four, and all matches were filtered with a false discovery rate < 0.05. A comparison between the AoVs’ decellularized fractions from the *wildtype* and *oim/oim* mice was then performed using the Proteome Discoverer consensus workflow on all identified unique peptide spectra. During the comparison, all samples were normalized to total peptide amount from each of the sample runs. Protein abundance was calculated using summed peptide abundances, and a protein ratio calculation was formulated in the software with a max fold change of 100 allowed. Imputation mode with low abundance resampling was applied to account for any missing values and a background-based *t*-test was applied to the protein abundances. For a complete list of the Proteome Discoverer processing and a consensus of workflow parameters used, see Supplemental File S1. Heat maps, volcano plots, and principal component analyses were created in Proteome Discoverer according to the user instructions. Gene ontology analysis (biological process (BP), cellular component (CC), and molecular function (MF)) [13] was performed through the DAVID platform (https://david.ncifcrf.gov (accessed on 12 January, 2023)) [14,15].

## 3. Results

### 3.1. Aortic Valves from oim/oim Mice Exhibit Temporal Disturbances in ECM Organization and Thickening

Osteogenesis imperfecta (OI) is a heritable connective tissue disorder caused by mutations in *COL1A1* or *COL1A2*, leading to defective collagen synthesis and ECM organization, and is primarily studied in skeletal tissue [8,12,16]. Interestingly, in addition to bone deformities, OI patients frequently develop heart valve dysfunction [17], predominantly affecting the AoV [18], yet how mutations in *COL1* negatively impact valvular performance is poorly understood. In mice, homozygous mutations in *Col1a2 (oim/oim)* phenocopy the human disease, and animals have been shown to develop structural AoV thickening at 9 months of age [11,19]. Here, we build on previous studies and further characterize the temporal progression of AoV disease in *oim/oim* mice using cellular, molecular, and functional assays.

**Figure 1:**
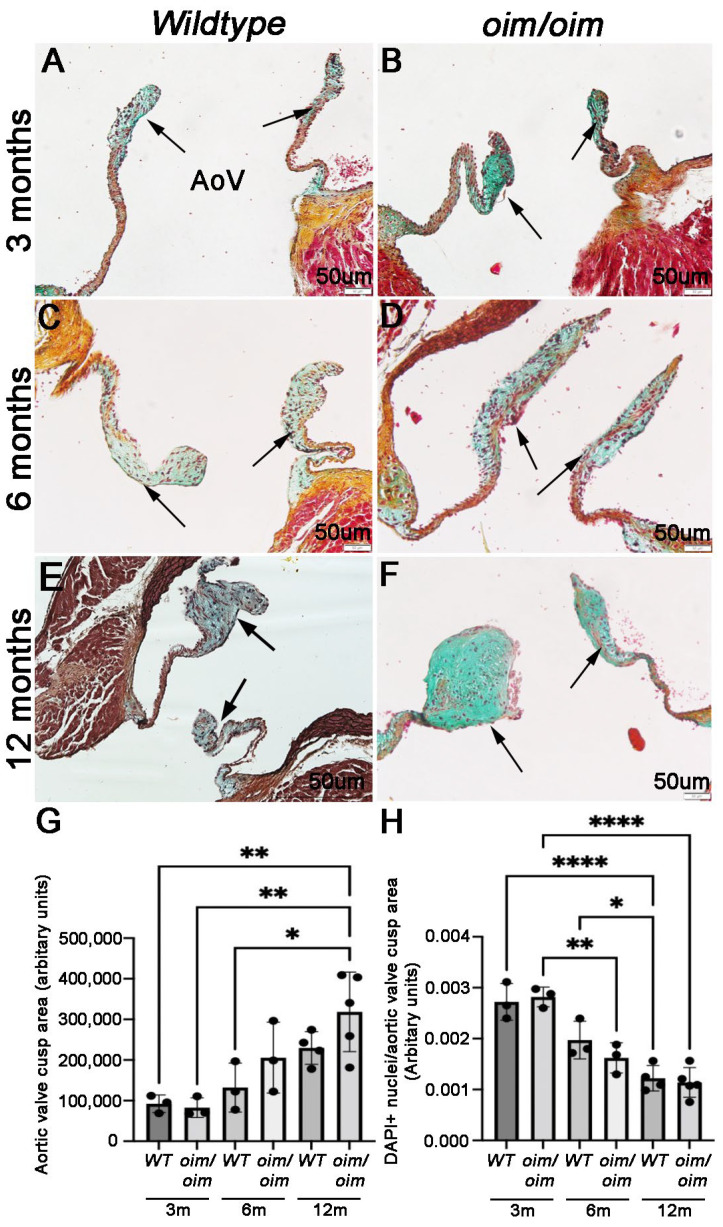
Aortic valve cusps from *oim/oim* mice thicken over time. Movat’s Pentachrome stain of aortic valve (AoV) regions from *wildtype* (*WT*) (**A**,**C**,**E**) and *oim/oim* (**B**,**D**,**F**) mice at 3 (**A**,**B**) (n = 3 for each genotype), 6 (**C**,**D**) (n = 3 for each genotype), and 12 (**E**,**F**) (n = 4 *WT*, n = 5 *oim/oim*) months. Arrows indicate AoV cusps. Elastic fibers (black); collagen fibers (yellow); mucin/proteoglycan (bright blue); fibrin (bright red); and muscle (red). (**G**) Quantitation of AoV cusp area based on 2D tissue sections as indicated. (**H**) The number of DAPI-positive nuclei normalized to AoV cusp area for each genotype and time point. Individual data points are plotted and represent biological replicates. * *p* < 0.05 ** *p* < 0.01, **** *p* < 0.0001 based on non-parametric 1-way ANOVA analysis. Note increased AoV cusp area in *oim/oim* mice at 12 months of age compared to 3 months.

To extend our understanding of the temporal progression of morphological changes in AoVs in OI, pentachrome staining was performed on tissue sections prepared from *oim/oim* (Figure 1B,D,F) and *wildtype* littermate control (Figure 1A,C,E) hearts at 3 (Figure 1A,B), 6 (Figure 1C,D), and 12 (Figure 1E,F) months of age. As suggested by histology (Figure 1A–F) and validated by quantitation in Figure 1G, trends of increased AoV cusp area were observed for both *wildtype* and *oim/oim* mice with aging, although AoV cusps were significantly thicker in *oim/oim* mice at 12 months compared to 3 months. One limitation of using the mouse model to study human disease is the significantly smaller size and, therefore, the tri-laminar structure of the AoV cusp being poorly distinguished [1]. Despite this, there is a notable gross increase in blue cytochemical staining in 12-month-old thickened cusps from *oim/oim* mice, which indicates abnormal levels of proteoglycan abundance. Interestingly, the number of DAPI-positive cells per area within each valve cusp did not increase with age; in fact, there was a notable decline at 12 months (Figure 1H), suggesting that the increase in AoV cusp area at 12 months of age is due to expansion of the ECM. It is worthy of mention that we note significant heterogeneity of the AoV thickening phenotype in the *oim/oim* experimental cohort at 6 and 12 months of age (see individual data plots in Figure 1G,H), which likely reflects variabilities in disease penetrance, similar to human disease. Consistent with the previous study by Cheek et al., other valves were not significantly affected by this mutation [11].

### 3.2. Oim/oim Mice Exhibit Cardiac Dysfunction by 12 Months of Age

**Figure 2:**
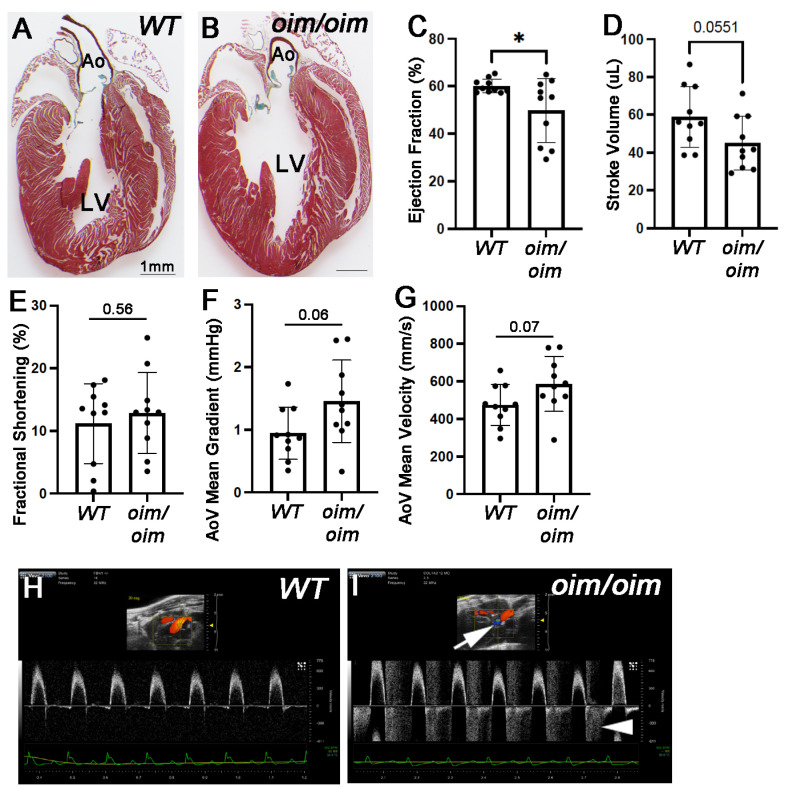
Cardiac function of *oim/oim* mice deteriorates by 12 months of age. (**A**,**B**) Movat’s pentachrome staining of whole heart sections from 12 month old *wildtype* (*WT*) (**A**) and *oim/oim* (**B**) mice. N = 9 for *WT*, n = 10 for *oim/oim*. Echocardiography findings of *WT* and *oim/oim* mice at 12 months of age including ejection fraction (**C**), stroke volume (**D**), fractional shortening (**E**), aortic valve (AoV) mean gradient (**F**) and AoV mean velocity (**G**). Individual data plots from each mouse are shown. * *p* < 0.05 based on non-parametric *t*-test analysis. (**H**,**I**) Representation of color doppler imaging from *WT* and *oim/oim* mice. Note retrograde blood flow in *oim/oim* mice (arrow and arrowhead) indicative of insufficiency observed in 5/10 *oim/oim* mice and 0/10 *WT* mice. Ao, aorta. LV, left ventricle.

AoVs from *oim/oim* become hyperplastic by 12 months of age (Figure 1F,G), although pentachrome-stained tissue sections of whole hearts indicate no other gross abnormalities in cardiac structure (Figure 2A,B). To determine if AoV defects observed in 12-month-old *oim/oim* mice function appropriately, echocardiography was performed. At 3 and 6 months of age, *oim/oim* mice do not exhibit significant cardiac dysfunction, consistent with normal AoV structure (Figure 1A–D) and findings previously reported at 9 months [11]. However, we built upon previous observations and performed echocardiography at later stages of disease (12 months) and reported decreases in ejection fraction (60.03 ± 2.9% vs. 49.64 ± 13.43%) (Figure 2C) and stroke volume (58.93 ± 16.09 µL vs. 45.03 ± 14.15 µL) (Figure 2D) in *oim/oim* mice, while fractional shortening was unchanged (Figure 2E). In addition, AoV mean gradient (0.95 ± 0.42 mmHg vs. 1.45 ± 0.66 mmHg) (Figure 2F) and mean velocity (474.54 ± 108.73 mm/s vs. 586.46 ± 146.14 mm/s) (Figure 2G) showed a trending increase in 12-month-old *oim/oim* mice. Consistent with histological findings, the severity of cardiac dysfunction was variable amongst the *oim/oim* cohort. Interestingly, using color doppler, we noted that 5/10 (50%) *oim/oim* mice developed regurgitation (Figure 2H,I), while 2/10 (20%) exhibited stenosis (peak velocity < 20 mm/Hg); both observations are reflected in data points in Figure 2F,G and likely contribute to the overall decreases in stroke volume and ejection fraction. In summary, gross AoV thickening during the late stages of osteogenesis imperfecta pathogenesis at least in mice, is associated with defects in valvular and cardiac function.

### 3.3. Changes in the ECM Proteome of Aortic Valves from oim/oim Mice Occurs Prior to Structure–Function Impairment

**Figure 3:**
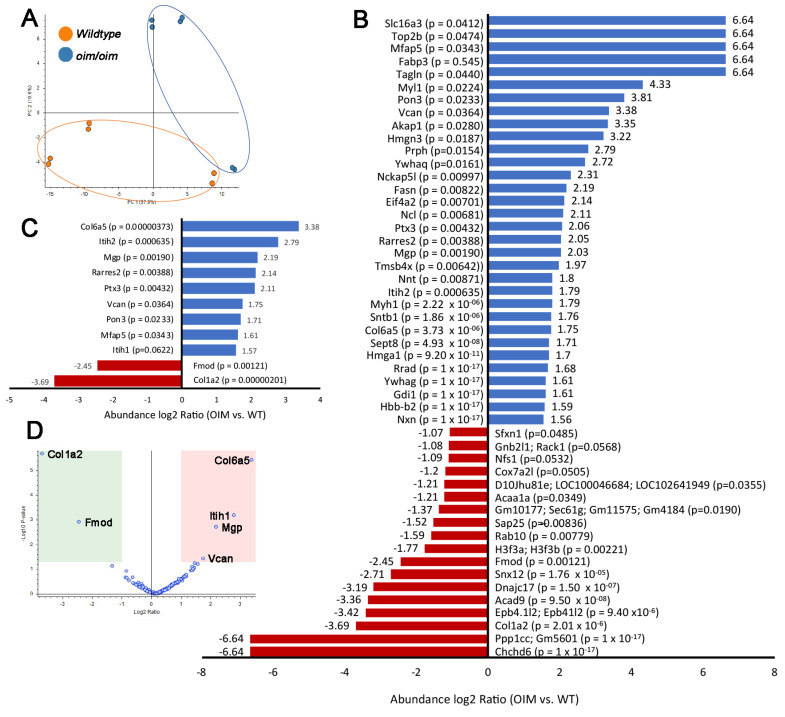
The ECM proteome is altered in *oim/oim* mice at 3 months of age. The ECM proteome is altered in *oim/oim* mice at 3 months of age. Individual AoV ring isolates (containing annulus ring and cusps) from n = 3 *wildtype* and *oim/oim* mice at 3 months of age were decellularized, enriched for the ECM, and subjected to liquid chromatography tandem mass spectrometry analysis. (**A**) Principal component analysis of all decellularized samples largely showed proteomic homology amongst genotypes, with biological variance observed for one biological replicate in each cohort. (**B**) Significantly altered proteins in *oim/oim* mice compared to *wildtype* controls (abundance ratio (sample/control) log2 fold change > 1 and *p*-value < 0.05. (**C**) Significantly altered proteins from (**B**) filtered for an association with the “ECM” based on gene ontology classification terms. (**D**) Volcano plot of differentially expressed proteins in AoVs from 3-month-old *oim/oim* mice versus *wildtype* (green, downregulated; red, upregulated). Fmod, fibromodulin; Itih1, inter-alpha-trypsin inhibitor heavy chain H2; Mgp, matrix gla protein; Vcan, versican.

Connective tissue disorders caused by mutations in ECM genes are commonly associated with disturbances in the organization of the ECM. In addition, ECM disturbances are characteristic of many forms of valve dysfunction [7]. To examine this further in osteogenesis-imperfecta-associated AoV pathogenesis, individual AoV rings (containing annulus and cusps) from 3-month-old mice (*oim/oim* and *wildtype* controls) were decellularized, enriched for the ECM fraction, and subjected to liquid chromatography tandem mass spectrometry (LC-MS/MS) analysis. This time point was chosen to detect early ECM proteomic changes within the AoV that underlie the progressive onset of structural and functional defects observed at 12 months. For this LC-MS/MS approach, experiments were performed for three biological replicates (each replicate including one AoV structure) for experimental duplicates. Principal component analysis (Figure 3A) of all decellularized samples largely shows proteomic homology amongst genotypes, although biological variance, as reflected in Figure 1 and Figure 2, is observed for one biological replicate in each cohort. Figure 3B indicates all (50) significantly altered proteins that passed the threshold criteria (abundance ratio (sample/control) > log2 fold change > 1, *p*-value < 0.05) in *oim/oim* mice compared to *wildtype* controls, that include 18 downregulated and 32 upregulated. Based on the protein abundance ratio between *oim/oim* versus *wildtype* samples, col1a2 was the most significantly downregulated protein (abundance ratio (log2) = −3.69); thereby confirming reduced protein in mice harboring a spontaneous mutation in the corresponding gene. From this list of 50 differentially expressed proteins, we filtered for “ECM”-associated proteins based on gene ontology (GO) classification terms, and we reported that 11/50 (22%) met the criteria (Figure 3C). Interestingly, reduced col1a2 was associated with a significant decrease in only one other protein, fibromodulin (abundance ratio (log2) = −2.45), and upregulation of nine other ECM proteins, including col6a5 (abundance ratio (log2) = 3.38), inter-alpha-trypsin inhibitor heavy chain H2 (abundance ratio (log2) = 2.79), matrix gla protein (abundance ratio (log2) = 2.19), and versican (abundance ratio (log2) 1.75). The corresponding volcano plot (Figure 3D) highlights the most differentially expressed proteins in *oim/oim* samples over *wildtype* controls.

**Figure 4:**
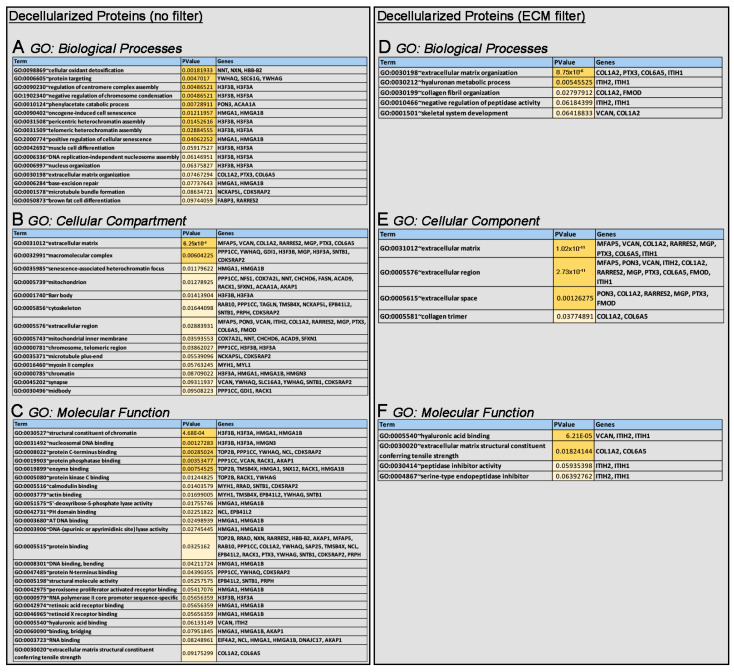
Gene ontology analysis of the altered ECM proteome in decellularized AoVs from *oim/oim* mice at 3 months of age. Gene ontology was performed using DAVID analysis on differentially expressed proteins as shown in Figure 3C (**A**–**C**) and those filtered for ECM association, as shown in Figure 3D (**D**–**F**). Outputs are reported as significantly affected GO classification terms related to biological processes (**A**,**D**), cellular compartments (**B**,**E**) and molecular functions (**C**,**F**). Dark yellow indicates *p*-value 0.05, light yellow indicates *p*-value < 0.01.

Additional GO analysis was performed on the 50 differentially expressed proteins found to better identify biological processes, cellular compartments, and molecular functions altered via loss of *Col1a2* and 49 other misregulated proteins. Affected biological processes include “cellular oxidant detoxification”, “protein targeting”, and “cell senescence” (Figure 4A), while molecular functions related to “structural constituent of chromatin”, “nucleosomal DNA binding”, and “protein C-terminus binding” were also noted (Figure 4C). As expected, “extracellular matrix” was significantly represented in affected cellular components, and additional GO analysis of ECM-filtered proteins (Figure 4D–F) identified more specific changes related to “extracellular matrix organization”, “hyaluronan metabolic processes”, “hyaluronan acid binding”, “extracellular matrix structural constituent conferring tensile strength”, “extracellular region”, and “extracellular space”. This LC-MS/MS analysis has identified early changes in the AoV ECM proteome at 3 months that precede structural and functional defects observed at 12 months.

### 3.4. Aortic Valves from oim/oim Mice Exhibit Disturbances in Proteoglycan Abundance from 3 Months of Age

**Figure 5:**
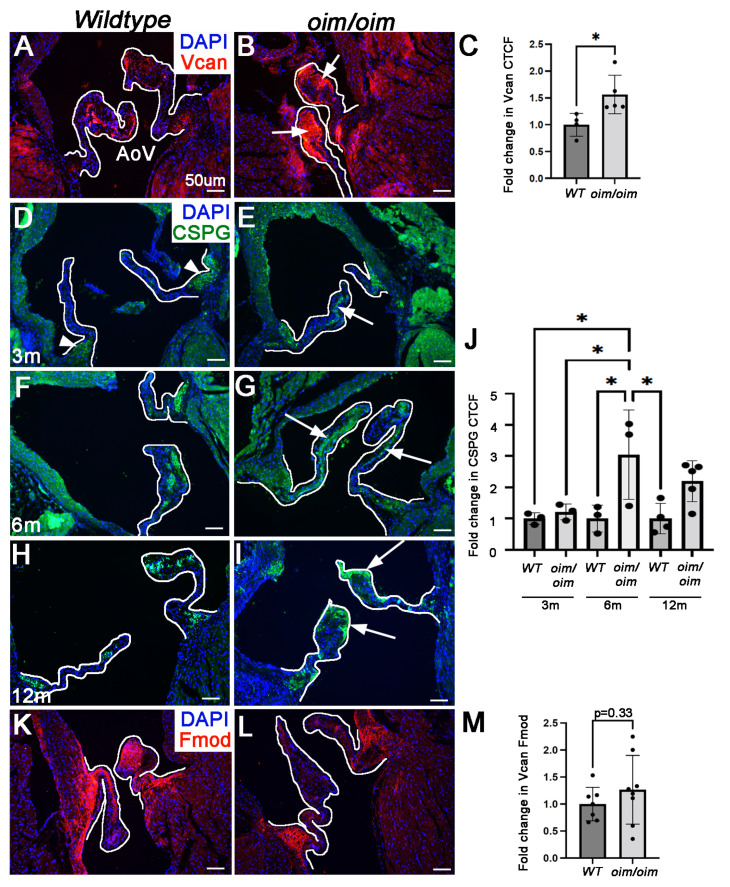
Proteoglycan abundance is altered in *oim/oim* mice from 3 months of age**.** Immunohistochemistry to detect versican (Vcan) (red, (**A**,**B**)), chondroitin sulfate proteoglycan (CSPG) (green, (**D**–**I**)), and fibromodulin (Fmod) (red, (**K**,**L**)) expression in aortic valve (AoV) regions from *wildtype* (*WT*) (**A**,**D**,**F**,**H**,**K**) and *oim/oim* (**B**,**E**,**G**,**I**,**L**) mice. Vcan and Fmod are shown at 3 months of age; CSPG at 3 (**D**,**E**), 6 (**F**,**G**), and 12 (**H**,**I**) months (n = 3 for each genotype and time point). DAPI (blue) indicate nuclei. (**C**,**J**,**M**) Corrected total cell fluorescence (CTCF) of Vcan (**C**) and CSPG (**J**) Fmod (**M**) in AoV regions normalized to area. * *p* < 0.05 based on non-parametric 1-way ANOVA analysis.

Proteomic analysis revealed differential changes and associated GO terms of ECM proteins, including proteoglycans such as versican (abundance ratio (log2) = 1.75) and fibromodulin (abundance ratio (log2) = −2.45), both of which have previously been shown to be critical components of the valve matrix (reviewed [7]). Versican is a member of the chondroitin sulfate family of proteoglycans (CSPGs) and is known to be critical for establishing and maintaining heart valve structure and function [7,20,21,22,23,24,25,26,27]. To validate LC-MS findings, we performed immunohistochemistry (IHC) and show a 1.5x-fold increase in versican that appears to be enriched at the distal tip of the AoV cusps (Figure 5A–C). To further determine if the overall expression of CSPG family members was altered in AoVs from *oim/oim* mice, tissue sections from mice at 3, 6, and 12 months of age were subjected to IHC to detect the peptide against the chondroitin chain of types A and B. As shown in Figure 5D–J, overall CSPG abundance was not affected at 3 months despite LC-MS/MS findings of individual CSPG family members, although ectopic expression was observed outside of the annular regions (as seen in *wildtypes* (arrowheads in Figure 5D)), within the core of the cusp (arrows, Figure 5E). In contrast, significant increases in CSPG expression were noted in *oim/oim* mice at 6 months of age throughout the cusp (arrows, Figure 5G,J). LC-MS/MS also revealed a significant decrease in fibromodulin, which has been shown to play a role in heart valve development [22], and while immunohistochemistry indicates similar reduction (Figure 5K,L), the difference was not significant (Figure 5M). Interestingly, the LC-MS/MS findings revealed significant increases in inter-alpha-trypsin inhibitors (ITI) H1 (Itih1) and H2 (Itih2) in AoVs from 3-month-old *oim/oim* mice (Figure 4). These proteins are known serine protease inhibitors involved in ECM stabilization and hyaluronic acid (HA) binding [28]. Roles for Itih1 and Itih2 have not been reported in the valve, but in arthritis, pathological degradation of cartilage has been attributed to ITI-HA complexes serving as substrates for ECM proteases—including ADAMTS-5 [29], which has been described as a key player in myxomatous valve disease [30]. Therefore, our findings warranted further validation. Together, these studies show that AoVs from *oim/oim* mice exhibit an imbalance in specific proteoglycan family members from 3 months of age.

### 3.5. Collagen Protein Types Are Differentially Expressed within the AoV Cusps in oim/oim Mice from 3 Months of Age

**Figure 6:**
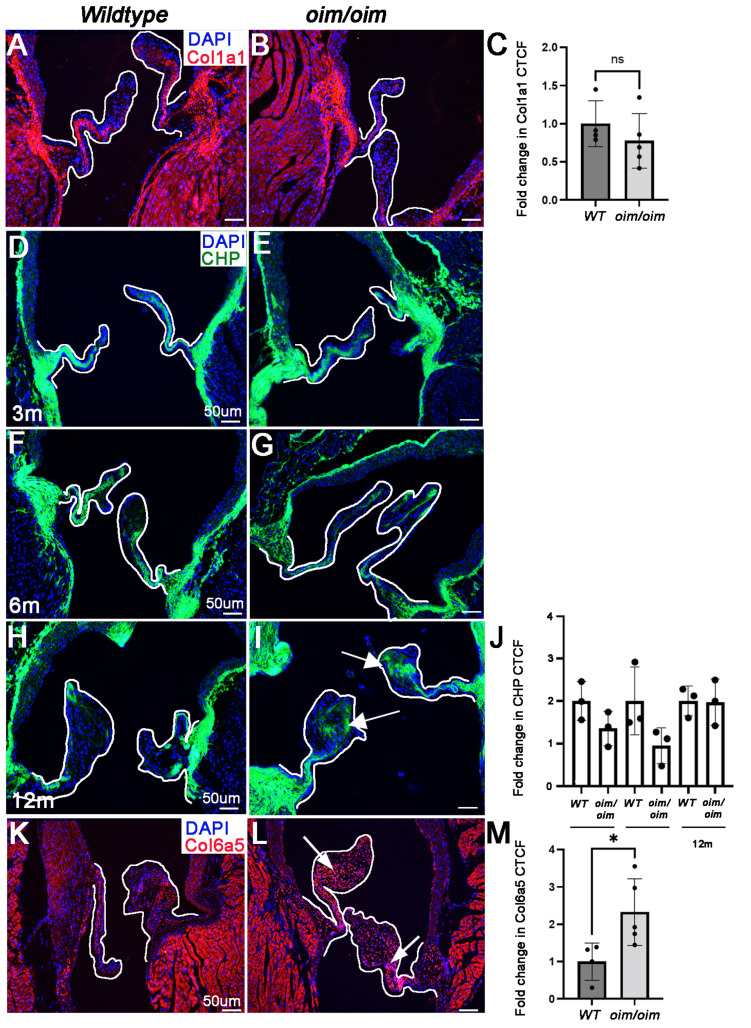
Collagen homeostasis is altered in aortic valve cusps during early stages of valve disease in 3 month old *oim/oim* mice. Immunocytochemistry to detect Col1a1 (red, (**A**,**B**)), Collagen Hybridization Peptide (CHP) (green) and Col6a5 (red, (**K**,**L**)) in aortic valve (AoV) regions from *wildtype* (*WT*) (**A**,**D**,**F**,**H**,**K**) and *oim/oim* (**B**,**E**,**G**,**I**,**L**) mice. Col1a1 and Col6a5 are shown at 3 months of age; CHP at 3 (**D**,**E**), 6 (**F**,**G**), and 12 (**H**,**I**) months. n = 3 for each genotype at each time point. Arrows in L indicate redistribution of CHP reactivity at the tip of the AoV cusp. DAPI (blue) indicate nuclei. (**C**,**J**,**M**) Corrected total cell fluorescence (CTCF) of Col1a1 (**C**), CHP (**J**), and Col6a5 (**M**) normalized to area. * *p* < 0.05 based on non-parametric 1-way ANOVA analysis.

Using LC-MS/MS, we observed that the underlying *Col1a2* mutation in *oim/oim* mice does not affect Col1a1 expression, as confirmed by immunohistochemistry (Figure 6A–C). To gain a better understanding of overall changes in collagen abundance in AoV cusps from *oim/oim* mice, we treated tissue sections from 3-, 6-, and 12-month-old mice with the GFP-tagged collagen-hybridizing peptide (CHP) that detects the localization of denatured collagen chains that typically serves as a biomarker for remodeled collagen. As shown in Figure 6D–J, the overall immunofluorescent reactivity of CHP was not significantly different over time or across genotypes, but we do note a redistribution of collagen towards the tip of the cusp in *oim/oim* mice at 12 months of age (Figure 6I). In contrast, LC-MS/MS did reveal a significant increase in Col6a5 in AoVs from *oim/oim* mice. Col6a5 is a member of the Col6 complex that acts as a cell-binding protein. As validated in Figure 6K–M, ectopic immunoreactivity of Col6a5 was observed throughout the enlarged cusp (arrows, Figure 6L). Together, these data highlight that in the absence of Col2a1, there are alterations in collagen homeostasis in *oim/oim* mice.

## 4. Discussion

Osteogenesis imperfecta (OI) is a rare (~11 per 100,000), inherited systemic connective tissue disease largely caused by mutations in the *COL1A1* or *COL1A2* genes [8,16]. A common phenotype is bone fragility, attributed to alterations in collagen content and organization that impair tissue stability and biomechanical function. Type I collagen is abundant in many tissues of high mechanical demand including the heart, and the incidence of valvulopathies, including valvular stenosis and regurgitation, in addition to thoracic aneurysms in inherited connective tissue disorders has been well described (reviewed [9]). However, despite the prevalence, the pathobiology of valvular phenotypes in these patients is poorly understood. While valvular dysfunction can be readily detected using standard imaging approaches (and anticipated in syndromic disorders), significant biomechanical impairment is often determined at late stages when non-interventional treatment options are limited. Therefore, in order to improve current therapeutic approaches, it is critical that the field determines early molecular and cellular events that lead to late biomechanical failure. To address this deficit, researchers have utilized the *oim/oim* mouse model of human disease to determine the effects of the *Col1a2* mutation on the structure–function relationships of many tissues affected by OI, although there are currently limited studies on the valvulopathy phenotype [11]. Here, we advance previous work and define the temporal evolution of aortic valvulopathy in *oim/oim* mice by correlating early proteomic changes with progressive alterations in ECM organization, AoV morphology, and cardiac function.

**Figure 7:**
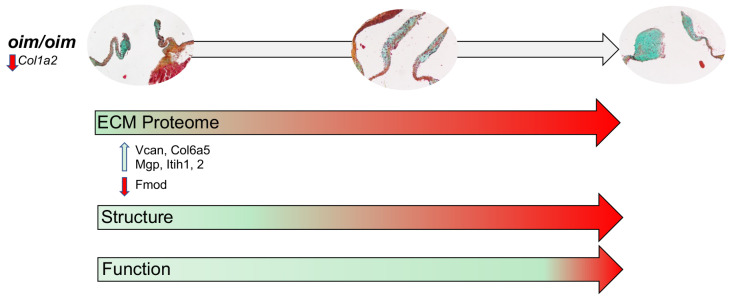
Summary of AoV valvuopathy in *oim/oim* mice. The temporal progression of AoV disease in *oim/oim* mice is initiated by mutations in *Col1a2,* which is hypothesized to lead to alterations in biochemical signals that are required to maintain physiological ECM homeostasis. As a result, secondary changes in the ECM proteome occur during early stages (3 months) of disease in *oim/oim* mice that, over time, cause structural deterioration (from 6 months onwards) and significant dysfunction by late stages (12 months).

ECM homeostasis is critical for maintaining heart valve function throughout life, and this is further highlighted by the reported incidence of valvulopathies in patients affected by hereditary connective tissue disorders. Our study has, for the first time, identified significant early changes in the decellularized valve proteome that likely contribute to the progressive functional impairment at later stages. As previously reported, more common OI phenotypes including bone fragility and muscle weakness (reviewed [31]) have largely been attributed to impaired synthesis of type I collagen, leading to defective tissue formation and maintenance. As expected, Col1a2 expression was significantly downregulated in AoVs from 3-month-old *oim/oim* mice compared to *wildtype*. However, in addition, we also note upregulation of the beaded filament Col6a5 at 3 months of age and re-distribution of denatured collagen towards the tip of the cusp at 12 months (Figure 6I), which are associated with changes in “collagen fiber organization”, “collagen trimer”, and “extracellular matrix structural constituent conferring tensile strength” GO classification terms (Figure 4). In parallel with collagen alterations, proteoglycan imbalance is also observed as increased versican, decreased Fibromodulin, increased Itih2 at 3 months, and enrichment of “hyaluronic acid binding” and “hyaluronic metabolic process”. This gross increase in proteoglycan abundance and disturbances in collagen homeostasis are consistent with features also described in surgical discards from degenerative valve disease patients [24,32,33]. It remains unclear how *Col1a2* mutations (and reduced expression) lead to secondary changes in the ECM that underlie structural deterioration and dysfunction in the *oim/oim* model. As Col1a1 and other fibrillar collagens are not increased to compensate for the loss of *Col1a2*, it is considered that the overall fibrillar collagen network is compromised in the *oim/oim* AoV cusp, which in turn negatively impacts critical biochemical signals required for physiological homeostasis. As a result, pathological ECM remodeling occurs (increased Vcan, Itih1, 2, altered collagen homeostasis), which is further exacerbated by aging and potentially influenced by biomechanical stresses (Figure 7). Worthy of mention, despite disruptions in ECM homeostasis, is that mechanical testing of AoVs from *oim/oim* mice showed no significant differences in radial or circumferential stiffness at 3 and 6 months (data not shown). Together, these data show that underlying OI-causing *Col1a2* mutations cause significant alterations in the contribution and organization of the AoV collagen and proteoglycan architecture, which progressively lead to structural thickening of the cusps and associated functional impairment.

Current treatment options for OI include prevention of bone fractures, control of symptoms and increase in bone masses through the administration of bisphosphonates, or pharmacological inhibition of osteoclast formation. Interestingly, therapeutic strategies have also been developed to target consequent molecular changes associated with the *Col1a2* mutation. This includes administration of romosozymab (a negative regulator of WNT signaling) as well as fresolimumab (an antibody that inhibits TGF-β). Both strategies have been shown to reduce collagen, while improving bone mass and strength (reviewed in [31]). Standardized treatment for OI-related valvulopathies is not as well developed. Moreover, current clinical management is limited to periodic surveillance of dysfunction, and only when the valve becomes functionally impaired is intervention recommended. This includes non-invasive transcatheter aortic valve replacement (TAVR) or more invasive measures of valve repair or replacement. Characterization of mouse models of human connective tissue disorders [34,35,36,37,38,39,40] has provided significant insights into the potential of therapeutic strategies for valvulopathies in this patient population. This has been most well developed in the mouse model of Marfan Syndrome induced via a C1039G substitution in the ECM gene *Fibrillin-1* (*Fbn1C1039G*), which develops mitral valve disease [34]. Using this model, the field has shown that inhibiting TGF-β signaling or preventing infiltration of circulatory monocytes can alleviate valvulopathies [34,37,41]. Comparable therapeutic studies for treating valvulopathies in OI are not as well developed. However, this study has identified early molecular and cellular changes in affected AoVs from *oim/oim* mice that likely underlie late functional defects, thereby providing a potential therapeutic window of opportunity and druggable processes/targets. Based on findings from this study, future work would benefit from studies exploring therapeutic strategies targeting the ECM to maintain or restore the trilaminar AoV cusp structure by attenuating progressive collagen disturbances and excess proteoglycan accumulation. However, these ECM components are abundant in many connective tissues, and therefore, valve-specific delivery would be required to avoid off-target effects. Despite this, the cancer field has made headway in therapeutically targeting the ECM to “fine-tune” the tumor environment to limit growth and migration. This includes exploring approaches to reduce versican secretion by targeting post translational modifications that lead to the production of disease-contributing isoforms [42] as well as treatment with the flavonoid extract baicalein to target MMP-2/9 expression and inhibit metastasis, potentially by preventing collagen degradation (reviewed in [43]). In the future, repurposing such cancer reagents in the treatment of valvulopathies warrants further investigation. In addition, findings from this study shed light on determining if high Itih2 levels serve as a biomarker for poor outcomes, similar to that described for other disease states (preeclampsia [44,45]), or mechanistically targeting reducing the inhibitory function of Itih2 to stabilize AoV ECM homeostasis.

This study has highlighted the temporal onset of AoV dysfunction in a mouse model of human OI at molecular, cellular, and functional levels. In taking this approach, we have gained a deeper understanding of how the Col2a1 mutation leads to a detrimental chain of events that causes an imbalance within the ECM niche, which in turn is exacerbated over time and underlies biomechanical failure. From here, we continue to explore more effective therapeutics in the treatment of valve dysfunction related to ECM disturbances.

## Figures and Tables

**Table 1:** Antibodies.

Antibody (α)	Raised In	Company, Product #	Dilution
Chondroitin Sulfate Proteoglycan (CSPG)	Mouse	Millipore Sigma, Burlington, MA, USA #C8035	1:200
VE Cadherin	Goat	R&D Systems, Minneapolis, MN, USA #AF1002	1:50
Actin, α-Smooth Muscle	Mouse	Millipore Sigma, Burlington, MA, USA #A2547	1:400
Col6a5	Mouse	Invitrogen, Waltham, MA, USA #PA5-70781	1:100
Fibromodulin	Rabbit	KeraFAST, Winston-Salem, NC, USA #ENH085-FP	1:100

## Data Availability

All protocols and data emanating from this study will be made available upon request to the senior and corresponding author. LC-MS/MS data are freely available in the Proteomics Identifications Database (PRIDE) data repository.

## Supplementary Materials

The following supporting information can be downloaded at: https://www.mdpi.com/article/10.3390/jcdd10080355/s1, Supplemental File 1: Proteome Discoverer processing and consensus of workflow parameters.
