# Supplementary material for: Temporal Progression of Aortic Valve Pathogenesis in a Mouse Model of Osteogenesis Imperfecta
Source: J Cardiovasc Dev Dis. 2023 Aug 20;10(8):355. doi: 10.3390/jcdd10080355 (PMC10455328; doi:10.3390/jcdd10080355)
Supplement: Supplementary file 1 [file jcdd-10-00355-s001.zip › jcdd-2533357-supplementary.pdf]

Supplementary Materials:
Supplemental File 1. Proteome Discoverer processing and consensus of workflow parameters.
Supplemental File 1: Proteome Discoverer Analysis Settings

==

Analysis Settings
==

==

Processing Step A: Workflow
==

Result name: 20120315_New_Decellularized_Analysis
Description: Processing workflow for precursor area quantification. Ion trap-detected HCD spectra using SequestHTwith Percolator validation. Specify the FASTA database and any additional modifications.
Workflow based on template: PWF_Tribrid_Precursor_Area_Quan_OT HCD_SequestHTnew_Percolator_mus
Creation date: 4/13/2021 4:31:51 PM
Created with Discoverer version: 2.4.1.15

The workflow tree:

```
 |-(0) Spectrum Files RC
   |-(1) Spectrum Selector
     |-(2) Sequest HT
       |-(3) Percolator
   |-(4) Minora Feature Detector
```

Processing node 0: Spectrum Files RC
----

1. Search Settings:
- File Name(s) (Hidden):
    C:\PDStudies\202103_Lincoln_OIMvalve_Project\20120315_C57_BR1_Decellularized.raw
    C:\PDStudies\202103_Lincoln_OIMvalve_Project\20120315_C57_BR1_Decellularized_rep.raw
    C:\PDStudies\202103_Lincoln_OIMvalve_Project\20120315_C57_BR2_Decellularized.raw
    C:\PDStudies\202103_Lincoln_OIMvalve_Project\20120315_C57_BR2_Decellularized_rep.raw
    C:\PDStudies\202103_Lincoln_OIMvalve_Project\20120315_C57_BR3_Decellularized.raw
    C:\PDStudies\202103_Lincoln_OIMvalve_Project\20120315_C57_BR3_Decellularized_rep.raw
    C:\PDStudies\202103_Lincoln_OIMvalve_Project\20120315_OIM_BR1_Decellularized.raw
    C:\PDStudies\202103_Lincoln_OIMvalve_Project\20120315_OIM_BR1_Decellularized_rep.raw
    C:\PDStudies\202103_Lincoln_OIMvalve_Project\20120315_OIM_BR2_Decellularized.raw
    C:\PDStudies\202103_Lincoln_OIMvalve_Project\20120315_OIM_BR2_Decellularized_rep.raw
    C:\PDStudies\202103_Lincoln_OIMvalve_Project\20120315_OIM_BR3_Decellularized.raw
    C:\PDStudies\202103_Lincoln_OIMvalve_Project\20120315_OIM_BR3_Decellularized_rep.raw
- Protein Database: Mus musculus (SwissProt TaxID=10090) (v2017-10-25)
- Enzyme Name: Trypsin (Full)
- Precursor Mass Tolerance: 20 ppm
- Fragment Mass Tolerance: 0.5 Da
- 1. Dynamic Modification: Carbamidomethyl / +57.021 Da (C)

2. Regression Settings:
- Regression Model: Non-linear Regression
- Parameter Tuning: Coarse

Processing node 1: Spectrum Selector

----
1. General Settings:
- Precursor Selection: Use MS1 Precursor
- Use Isotope Pattern in Precursor Reevaluation: True
- Provide Profile Spectra: Automatic

2. Spectrum Properties Filter:
- Lower RT Limit: 0
- Upper RT Limit: 0
- First Scan: 0
- Last Scan: 0
- Lowest Charge State: 0
- Highest Charge State: 0
- Min. Precursor Mass: 350 Da
- Max. Precursor Mass: 5000 Da
- Total Intensity Threshold: 0
- Minimum Peak Count: 1

3. Scan Event Filters:
- MS Order: Is Not MS1
- Min. Collision Energy: 0
- Max. Collision Energy: 1000
- Scan Type: Is Full

4. Peak Filters:
- S/N Threshold (FT-only): 1.5

5. Replacements for Unrecognized Properties:
- Unrecognized Charge Replacements: Automatic
- Unrecognized Mass Analyzer Replacements: ITMS
- Unrecognized MS Order Replacements: MS2
- Unrecognized Activation Type Replacements: CID
- Unrecognized Polarity Replacements: +
- Unrecognized MS Resolution@200 Replacements: 60000
- Unrecognized MSn Resolution@200 Replacements: 30000

6. Precursor Pattern Extraction:
- Precursor Clipping Range Before: 2.5 Da
- Precursor Clipping Range After: 5.5 Da

Processing node 2: Sequest HT
----
1. Input Data:
- Protein Database: Mus musculus (SwissProt TaxID=10090) (v2017-10-25)
- Enzyme Name: Trypsin (Full)
- Max. Missed Cleavage Sites: 4
- Min. Peptide Length: 4
- Max. Peptide Length: 144
- Max. Number of Peptides Reported: 10

2. Tolerances:
- Precursor Mass Tolerance: 20 ppm
- Fragment Mass Tolerance: 0.05 Da
- Use Average Precursor Mass: False
- Use Average Fragment Mass: False

3. Spectrum Matching:
- Use Neutral Loss a Ions: True
- Use Neutral Loss b Ions: True
- Use Neutral Loss y Ions: True

- Use Flanking Ions: True
- Weight of a Ions: 0
- Weight of b Ions: 1
- Weight of c Ions: 0
- Weight of x Ions: 0
- Weight of y Ions: 1
- Weight of z Ions: 0

4. Dynamic Modifications:
- Max. Equal Modifications Per Peptide: 3
- Max. Dynamic Modifications Per Peptide: 4
- 1. Dynamic Modification: Oxidation / +15.995 Da (M)
- 2. Dynamic Modification: Carbamidomethyl / +57.021 Da (C)
- 3. Dynamic Modification: Acetyl / +42.011 Da (K, R)

6. Dynamic Modifications (protein terminus):
- 1. N-Terminal Modification: Acetyl / +42.011 Da (N-Terminus)
- 2. N-Terminal Modification: Met-loss / -131.040 Da (M)
- 3. N-Terminal Modification: Met-loss+Acetyl / -89.030 Da (M)

Processing node 3: Percolator
----
1. Target/Decoy Strategy:
- Target/Decoy Selection: Concatenated
- Validation based on: q-Value

2. Input Data:
- Maximum Delta Cn: 0.05
- Maximum Rank: 0

3. FDR Targets:
- Target FDR (Strict): 0.01
- Target FDR (Relaxed): 0.05

Processing node 4: Minora Feature Detector
----
1. Peak & Feature Detection:
- Min. Trace Length: 5
- Max. ΔRT of Isotope Pattern Multiplets [min]: 0.2

2. Feature to ID Linking:
- PSM Confidence At Least: High

==
Consensus Step : Workflow
==
Result name: 20120315_New_Decellularized_Analysis
Description: Result filtered for high confident peptides, with enhanced peptide and protein annotations. Add FASTA file with common contaminants to the Protein Marker node. Quan abunaces are normalized to the same total peptide amount per channel and scaled, so that the average abunce per protein and peptide is 100.
Workflow based on template: CWF_Comprehensive_Enhanced Annotation_LFQ_and_Precursor_Quan_relaxed-SN3
Creation date: 4/13/2021 4:31:56 PM
Created with Discoverer version: 2.4.1.15

The workflow tree:

 |-(0) MSF Files
  |-(1) PSM Grouper
   |-(2) Peptide Validator
    |-(3) Peptide and Protein Filter
     |-(4) Protein Scorer
      |-(5) Protein Grouping
       |-(6) Peptide in Protein Annotation
      |-(7) Protein FDR Validator
     |-(8) Protein Annotation
     |-(9) Protein Marker
  |-(10) Feature Mapper
   |-(11) Precursor Ions Quantifier

Post-processing nodes:

 |-(12) Result Statistics
 |-(13) Display Settings
 |-(14) Data Distributions

Processing node 0: MSF Files
----
1. Storage Settings:
- Spectra to Store: Identified or Quantified
- Feature Traces to Store: All

2. Merging of Identified Peptide and Proteins:
- Merge Mode: Globally by Search Engine Type

3. FASTA Title Line Display:
- Reported FASTA Title Lines: Best match
- Title Line Rule: standard

4. PSM Filters:
- Maximum Delta Cn: 0.05
- Maximum Rank: 0
- Maximum Delta Mass: 0 ppm

Hidden Parameters:
- MSF File(s): C:\PDStudies\202103_Lincoln_OIMvalve_Project\202103_Lincoln_OIMvalveProject\20120315_New_Decellularized_Analysis.msf

----
Processing node 1: PSM Grouper
----
1. Peptide Group Modifications:
- Site Probability Threshold: 75

Processing node 2: Peptide Validator
----
1. General Validation Settings:
- Validation Mode: Automatic (Control peptide level error rate if possible)
- Target FDR (Strict) for PSMs: 0.01
- Target FDR (Relaxed) for PSMs: 0.05
- Target FDR (Strict) for Peptides: 0.01

- Target FDR (Relaxed) for Peptides: 0.05

2. Specific Validation Settings:
- Validation Based on: q-Value
- Target/Decoy Selection for PSM Level FDR Calculation Based on Score: Automatic
- Reset Confidences for Nodes without Decoy Search (Fixed score thresholds): False

----
Processing node 3: Peptide and Protein Filter
----
1. Peptide Filters:
- Peptide Confidence At Least: Medium
- Keep Lower Confident PSMs: True
- Minimum Peptide Length: 4
- Remove Peptides Without Protein Reference: False

2. Protein Filters:
- Minimum Number of Peptide Sequences: 1
- Count Only Rank 1 Peptides: False
- Count Peptides Only for Top Scored Protein: False

Processing node 4: Protein Scorer

No parameters

Processing node 5: Protein Grouping
----
1. Protein Grouping:
- Apply strict parsimony principle: True

Processing node 6: Peptide in Protein Annotation
----
1. Flanking Residues:
- Annotate Flanking Residues of the Peptide: True
- Number Flanking Residues in Connection Tables: 1

2. Modifications in Peptide:
- Protein Modifications Reported: Only for Master Proteins

3. Modifications in Protein:
- Modification Sites Reported: All And Specific
- Minimum PSM Confidence: High
- Report Only PTMs: True

4. Positions in Protein:
- Protein Positions for Peptides: Only for Master Proteins

Processing node 7: Protein FDR Validator
----
1. Confidence Thresholds:
- Target FDR (Strict): 0.01
- Target FDR (Relaxed): 0.05

Processing node 8: Protein Annotation
----
1. Annotation Aspects:

- 1. Aspect: Biological Process
- 2. Aspect: Cellular Component
- 3. Aspect: Molecular Function
- 4. Aspect: None
- 5. Aspect: None
- 6. Aspect: None

Processing node 9: Protein Marker
----
5. Annotate Species:
- As Species Map: False
- As Species Names: False

6. Mark Additional Entities:
- Annotation Groups: False
- Pathway Groups: False
- Modification Sites: True
- Peptide Isoform Groups: True

Processing node 10: Feature Mapper
----
1. Chromatographic Alignment:
- Perform RT Alignment: True
- Maximum RT Shift [min]: 10
- Mass Tolerance: 10 ppm
- Parameter Tuning: Coarse

2. Feature Linking and Mapping:
- RT Tolerance [min]: 0
- Mass Tolerance: 0 ppm
- Min. S/N Threshold: 0

Processing node 11: Precursor Ions Quantifier
----
1. General Quantification Settings:
- Peptides to Use: Unique + Razor
- Consider Protein Groups for Peptide Uniqueness: True
- Use Shared Quan Results: True
- Reject Quan Results with Missing Channels: False

2. Precursor Quantification:
- Precursor Abundance Based On: Intensity
- Min. # Replicate Features [%]: 0

3. Normalization and Scaling:
- Normalization Mode: Total Peptide Amount
- Scaling Mode: On All Average

4. Exclude Peptides from Protein Quantification:
- For Normalization: Use All Peptides
- For Protein Roll-Up: Use All Peptides
- For Pairwise Ratios: Exclude Modified

5. Quan Rollup and Hypothesis Testing:
- Protein Abundance Calculation: Summed Abundances
- N for Top N: 3
- Protein Ratio Calculation: Pairwise Ratio Based
- Maximum Allowed Fold Change: 100

- Imputation Mode: None
- Hypothesis Test: t-test (Background Based)

6. Quan Ratio Distributions:
- 1st Fold Change Threshold: 2
- 2nd Fold Change Threshold: 4
- 3rd Fold Change Threshold: 6
- 4th Fold Change Threshold: 8
- 5th Fold Change Threshold: 10

Processing node 12: Result Statistics

No parameters

Processing node 13: Display Settings
----
1. General:
- Filter Set:

    ###   Filter Set MasterProteinFilter.filterset contains the following filters:
    ###   Row Filter for TargetProtein:
    ###   Master is equal to Master
    ###

'magellan filter set' 1 'MasterProteinFilter.filterset' FiltersetProperties 1 'LastFileName' 'C:\Users\frank.berg\Desktop\MasterProteinFilter.filterset' Filter 'TargetProtein' 1 NARY_AND 1 = FilterConditionProperties 1 'NamedComparableFilterCondition/DisplayPropertyHint' 'Master' property 'Thermo.PD.EntityDataFramework.MasterProteinAssessment, Thermo.Magellan.EntityDataFramework' 'IsMasterProtein' constant 'Thermo.PD.EntityDataFramework.MasterProteinAssessment, Thermo.Magellan.EntityDataFramework' 'IsMasterProtein'

===
Consensus Step Validation
===
Result name: 20120315_New_Decellularized_Analysis
Description: Result filtered for high confident peptides, with enhanced peptide and protein annotations. Add FASTA file with common contaminants to the Protein Marker node. Quan abunaces are normalized to the same total peptide amount per channel and scaled, so that the average abunce per protein and peptide is 100.
Workflow based on template: CWF_Comprehensive_Enhanced Annotation_LFQ_and_Precursor_Quan_relaxed-SN3
Creation date: 4/13/2021 4:31:56 PM
Created with Discoverer version: 2.4.1.15

Peptide Validator nodes:

Processing node 2: Peptide Validator
----
1. General Validation Settings:
- Validation Mode: Automatic (Control peptide level error rate if possible)
- Target FDR (Strict) for PSMs: 0.01
- Target FDR (Relaxed) for PSMs: 0.05
- Target FDR (Strict) for Peptides: 0.01

- Target FDR (Relaxed) for Peptides: 0.05

2. Specific Validation Settings:
- Validation Based on: q-Value
- Target/Decoy Selection for PSM Level FDR Calculation Based on Score: Automatic
- Reset Confidences for Nodes without Decoy Search (Fixed score thresholds): False

Additional information:

Used validation mode: 'Automatic (Control peptide level error rate if possible)'.
All PSMs have PEPs. Qvality will be used for peptides.
Updated peptide confidences using qvality.

Protein Validator nodes:

Processing node 7: Protein FDR Validator
----
1. Confidence Thresholds:
- Target FDR (Strict): 0.01
- Target FDR (Relaxed): 0.05

==
Processing Step A: Validation
==
Result name: 20120315_New_Decellularized_Analysis
Result file: C:\PDStudies\202103_Lincoln_OIMvalve_Project\202103_Lincoln_OIMvalveProject\20120315_New_Decellularized_Analysis.msf
Description: Processing workflow for precursor area quantification. Ion trap-detected HCD spectra using SequestHTwith Percolator validation. Specify the FASTA database and any additional modifications.
Workflow based on template: PWF_Tribrid_Precursor_Area_Quan_OT HCD_SequestHTnew_Percolator_mus
Creation date: 4/13/2021 4:31:51 PM
Created with Discoverer version: 2.4.1.15

Psm Validator nodes:

Processing node 3: Percolator
----
1. Target/Decoy Strategy:
- Target/Decoy Selection: Concatenated
- Validation based on: q-Value

2. Input Data:
- Maximum Delta Cn: 0.05
- Maximum Rank: 0

3. FDR Targets:
- Target FDR (Strict): 0.01
- Target FDR (Relaxed): 0.05

Validation for Processing Node: Sequest HT (2)

Percolator Output:

Results for Sequest HT (2):

Iteration 1:	Estimated 17989 PSMs with q<0.01
Iteration 2:	Estimated 28312 PSMs with q<0.01
Iteration 3:	Estimated 32642 PSMs with q<0.01
Iteration 4:	Estimated 34325 PSMs with q<0.01
Iteration 5:	Estimated 35492 PSMs with q<0.01
Iteration 6:	Estimated 35817 PSMs with q<0.01
Iteration 7:	Estimated 36014 PSMs with q<0.01
Iteration 8:	Estimated 36000 PSMs with q<0.01
Iteration 9:	Estimated 36076 PSMs with q<0.01
Iteration 10:	Estimated 36066 PSMs with q<0.01

Learned normalized SVM weights for the 3 cross-validation splits:

Split1	Split2	Split3	FeatureName
0.0798	-0.0085	0.0491	XCorr
0.2026	0.2049	0.2273	Delta Cn From Second PSM
1.2600	1.5484	1.3428	Binomial Score
-0.3721	-0.4406	-0.4221	Isolation Interference [%]
-0.2304	-0.2181	-0.4164	MH+ [Da]
0.6353	2.3288	0.9372	Delta Mass [Da]
0.3888	-0.6898	0.4365	Delta Mass [ppm]
-1.1355	0.0818	1.1024	Absolute Delta Mass [Da]
-5.0238	-8.6499	-7.9411	Absolute Delta Mass [ppm]
0.2569	0.2469	0.3857	Peptide Length
0.0000	0.0000	0.0000	Is z=1
-0.2940	-0.3761	-0.2998	Is z=2
0.1964	0.2429	0.2040	Is z=3
0.1682	0.2364	0.1850	Is z=4
0.1151	0.1607	0.0818	Is z=5
0.1299	0.1350	0.1181	Is z>5
-1.3697	-1.6907	-1.5056	# Missed Cleavages
0.0263	0.0609	0.0075	Log Peptides Matched
0.5587	0.7057	0.5429	Log Total Intensity
0.2410	0.2507	0.2310	Fraction Matched Intensity [%]
-0.7115	-0.8580	-0.8230	Fragment Coverage Series A, B, C [%]
-0.1624	-0.2024	-0.1853	Fragment Coverage Series X, Y, Z [%]
-0.3604	-0.4170	-0.3223	Log Matched Fragment Series Intensities A, B, C
-0.4408	-0.5106	-0.3823	Log Matched Fragment Series Intensities X, Y, Z
0.0705	0.0521	0.1017	Longest Sequence Series A, B, C
0.0323	0.0129	0.0352	Longest Sequence Series X, Y, Z
0.1638	0.0668	0.1481	IQR Fragment Delta Mass [Da]
-0.1203	-0.1763	-0.1065	IQR Fragment Delta Mass [ppm]
-0.2419	-0.1056	-0.2392	Mean Fragment Delta Mass [Da]
0.0805	0.0072	0.0535	Mean Fragment Delta Mass [ppm]
0.3097	0.4030	0.3582	Mean Absolute Fragment Delta Mass [Da]
-0.3768	-0.2851	-0.3753	Mean Absolute Fragment Delta Mass [ppm]
-13.9003	-18.5101	-15.8022	m0

==
Consensus Step Quantification
==
Result name: 20120315_New_Decellularized_Analysis
Description: Result filtered for high confident peptides, with enhanced peptide and protein annotations. Add FASTA file with common contaminants to the Protein Marker node. Quan abunaces are normalized to the same total peptide amount per channel and scaled, so that the average abunce per protein and peptide is 100.
Workflow based on template: CWF_Comprehensive_Enhanced Annotation_LFQ_and_Precursor_Quan_relaxed-SN3
Creation date: 4/13/2021 4:31:56 PM
Created with Discoverer version: 2.4.1.15

Processing node 11: Precursor Ions Quantifier
----
1. General Quantification Settings:
- Peptides to Use: Unique + Razor
- Consider Protein Groups for Peptide Uniqueness: True
- Use Shared Quan Results: True
- Reject Quan Results with Missing Channels: False

2. Precursor Quantification:
- Precursor Abundance Based On: Intensity
- Min. # Replicate Features [%]: 0

3. Normalization and Scaling:
- Normalization Mode: Total Peptide Amount
- Scaling Mode: On All Average

4. Exclude Peptides from Protein Quantification:
- For Normalization: Use All Peptides
- For Protein Roll-Up: Use All Peptides
- For Pairwise Ratios: Exclude Modified

5. Quan Rollup and Hypothesis Testing:
- Protein Abundance Calculation: Summed Abundances
- N for Top N: 3
- Protein Ratio Calculation: Pairwise Ratio Based
- Maximum Allowed Fold Change: 100
- Imputation Mode: None
- Hypothesis Test: t-test (Background Based)

6. Quan Ratio Distributions:
- 1st Fold Change Threshold: 2
- 2nd Fold Change Threshold: 4
- 3rd Fold Change Threshold: 6
- 4th Fold Change Threshold: 8
- 5th Fold Change Threshold: 10
